# Tensile Test Coupled with an EBSD Study of a GH4169 Ring Rolled Product

**DOI:** 10.3390/ma15082891

**Published:** 2022-04-14

**Authors:** Hao Wang, Haoyi Niu, Hao Wu, Rengeng Li, Guohua Fan

**Affiliations:** 1 Key Laboratory for Light-Weight Materials, College of Materials Science and Engineering, Nanjing Tech University, Nanjing 210009, China; haow@njtech.edu.cn (H.W.); hwu@njtech.edu.cn (H.W.); 2 College of Materials Science and Engineering, Chongqing University, Chongqing 400030, China; hyniu@cqu.edu.cn

**Keywords:** ring rolling, in situ, ebsd, tensile test, slip system

## Abstract

An in situ tensile test of the ring-rolled GH4169 alloy is performed to investigate the plastic deformation behavior at the micro level. Slip system activations are identified by slip traces captured by a scanning electron microscope and lattice orientation data acquired by electron backscattered diffraction. Our results demonstrated that the fraction of low-angle grain boundaries gradually increased upon tensile deformation, and the misorientation evolution in the grain interior was severely inhomogeneous. The Schmid factors at the grains of interest are calculated for comparison with the actual activated slip systems. Most of the slip system activation coincides with the Schmid law, as opposed to the initiation of other potential slip systems at some grains.

## 1. Introduction

Ni-based superalloys, owing to their excellent mechanical and chemical properties at elevated temperatures, have been widely applied in the aerospace, marine, nuclear reactor, and chemical industries [1,2]. Usually, the structural components with high strength and high toughness have poor machinability inevitability [3,4]. To avoid the machinability problem, the processing steps of fabricating superalloys should be reduced as much as possible. Ring rolling, an incremental bulk metal forming process, has been identified as one of the most promising processes for the fabrication of seamless ring components because of its simplicity [5]. Ring rolling is fit to fabricate various products with a wide variety of sizes and shapes, which is widely applied in automotive, bearing, or aerospace industries [6]. Compared to the counterparts subjected to the traditional roll bending and welding process, the products subjected to the ring rolling have a substantially higher processing precision and an outstanding mechanical performance. The application of the ring rolling eliminates the welding process, and thus reduces both cost and resource consumption. However, ring-rolled products, especially the hot ring-rolled products, often exhibit a strong texture and heterogeneous secondary phase distributions [7,8]. The anisotropy of microstructures will degrade the service performance of ring-rolled products. Usually, the deformation temperature and rolling parameters determine the microstructures and the mechanical properties of the final ring-rolled products. Numerous attempts have been conducted to optimize the ring rolling parameters [9]. The flow behavior and microstructure evolution during hot ring rolling are the main research focus [10,11]. By controlling radial flow of metal during hot ring rolling, the reasonable fibrous macrostructure was obtained, and thus improved the mechanical properties [7]. The microstructure evolution during hot ring rolling was investigated using finite element simulation and analyzed in terms of both simulations and experiments [10,11,12,13]. Based on the simulation results, many appropriate methods and rolling parameters have been developed to meet the demands of high-performance component [14,15]. Some post treatment after ring rolling have been conducted to further reduce the residual stress of ring-rolled products [16]. However, little attention has been paid on the mechanical and microstructural evolutions of ring-rolled products during in situ tensile.

Scanning electron microscope (SEM) equipped with electron backscattered diffraction (EBSD) detector and in situ tensile equipment is deemed to be a powerful tool for tracking the evolution of texture and microstructures in the micro-region of interest during plastic deformation [17,18,19], making it possible to investigate the deformation mechanism deeply. A fundamental and practical research on EBSD orientation and collection has been elucidated by Britton [20]. Xia [21] revealed the local stress effected strength-ductility synergy of layered aluminum. Gao et al. [22] investigated the evolution of annealing twins and their interaction with dislocations in an Inconel 625 alloy. Chen et al. [23] analyzed the slip trace and rotation behavior of aluminum alloy during tensile deformation, and several theoretical rotation directions were compared and examined with the observed rotation directions. The crack initiation and propagation of Ni-based superalloys with cooling holes has been investigated by Li et al. [24] The twinning-detwinning behavior of sheet Mg has been well explained with in situ SEM-EBSD by Choi et al. [25]. However, few in situ investigations were conducted on the evolution of the texture and microstructure of the ring-rolled GH4169 alloy. The deformation mechanism of the GH4169 alloy subjected to the ring rolling remains unclear.

In this study, a GH4169 alloy, as a precipitation-strengthened nickel-based superalloy, was subjected to the ring rolling. In situ tensile tests using SEM-EBSD were performed to investigate the deformation mechanism of the ring-rolled GH4169 alloy. The microstructure evolution of the ring-rolled GH4169 alloy subjected to various strains were investigated. The evolutions of grain boundary, misorientation and dislocation density were discussed in details.

## 2. Materials and Test Method

### 2.1. Specimen Preparation

**Figure 1:**
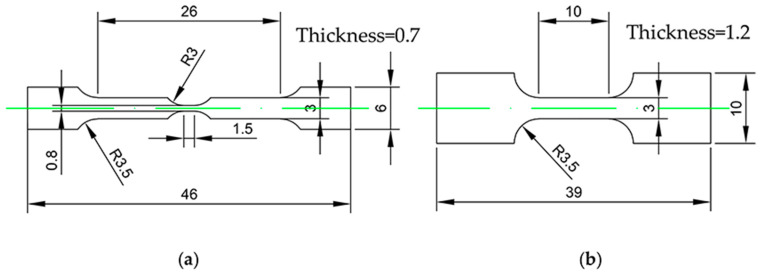
Geometry of the tensile test specimens. (**a**) In situ test specimen, (**b**) ex situ test specimen. Units, mm.

The commercial bearing ring was brought from Wuxi Parker New Material Technology Co, Ltd. (Wuxi, China) Tensile specimens were cut from the bearing ring along circumferential direction (CD) and radial direction (RD) using electrical discharge machining (EDM) to investigate the mechanical anisotropy. Figure 1 depicted the geometry of the in situ and the ex situ tensile specimens. The tensile specimen geometry is derived from Nautiyal P. et al. [26] and Zhang et al. [27].

For EBSD characterization, the observation surface was grounded with sandpaper up to 1500# grit and then electrochemically polished to a mirror-like gloss. The electrochemical-polishing section was carried out at room temperature in a 20 V constant voltage mode for 5 s, using a solution containing 10% perchloric acid and 90% ethanol.

### 2.2. Test Method

Microstructure observations were conducted using a TESCAN Clara scanning electron microscope (SEM). The coupled EBSD data were collected on an EDAX velocity camera series through a Texture & Elemental Analytical Microscopy (TEAM, San Diego, CA, USA) software. The parameters set for EBSD data collection on TEAM are as follows, binning 4 × 4, standard mode and adjusted exposure time to near 1000 frames per second (in this study, about 998 frames per second). The TSL database nickel (m-3m) was selected for pattern indexing. The in situ tensile test was performed on a Mini-MTS tensile stage installed in a TESCAN Clara chamber. In situ tensile tests were interrupted at yielding, and strain levels of 12%, 18%, and 24% after yielding for SEM-EBSD data capture. The strain levels are performed above elastic limit, the corresponding true stress–strain system is controlled by grips displacement, and further validated by post stress–strain data processing. In situ EBSD data were collected at an accelerating voltage of 20 KV, working distance of 15 mm and a current of 20 nA, and the step size was set to 0.15 μm. An open-source Mtex [28] toolbox was employed for postprocessing of EBSD data. Combined SEM data was captured under a current of 1 nA for better imaging of slip traces.

A conventional uniaxial tensile test was carried out on an Instron 5895 tensile test equipment at room temperature. The strain rates were both set to be 1 × 10^−3^ s^−1^ for conventional and in situ tensile tests. Samples were tensile-tested three times under the same condition. As the limits of sample size and low stiffness of Mini-MTS machine, the computed stress-strain curve may not be fully transported to macroscopic tensile test [29].

## 3. Result and Discussion

**Figure 2:**
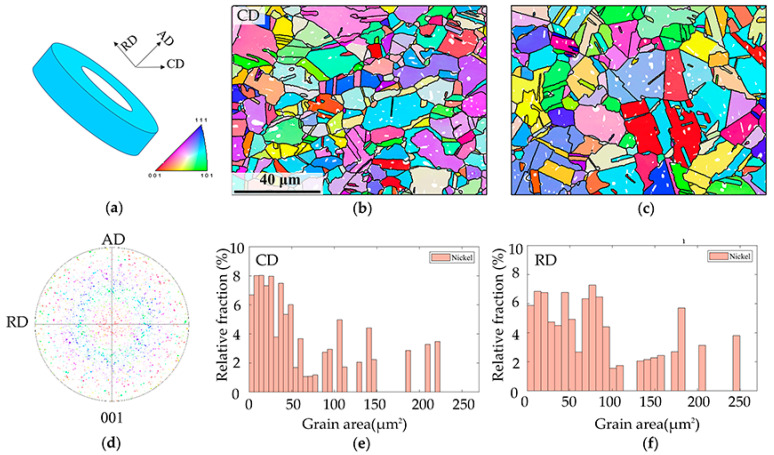
(**a**) Sample orientation. (**b**) EBSD mapping along CD. (**c**) EBSD mapping along RD. (**d**) Pole figure of CD. (**e**) Grain size distribution of CD. (**f**) Grain size distribution of RD.

The microstructure and orientation of the GH4169 alloy are shown in Figure 2. The microstructure of the observation surface reveals a relatively random orientation distribution, different from the traditional rolled products with strong texture. The microstructure of the ring-rolled GH4169 alloy features a secondary phase with no obvious anisotropic distribution and a considerable number of twins. The secondary phase, which mainly consisted of γ’, was carefully separated by a confidence index (CI) threshold. In this study, we will focus on the deformation mechanism, and thus the effects of the homogeneous secondary phase will not be discussed further. The twins generally show a Σ3 grain boundary, with approximately 60° of misorientation in the current specimens. The grain size distribution of the ring-rolled alloy is even, with an average grain size of 20 μm. The shapes of the grains are nearly equiaxed.

**Figure 3:**
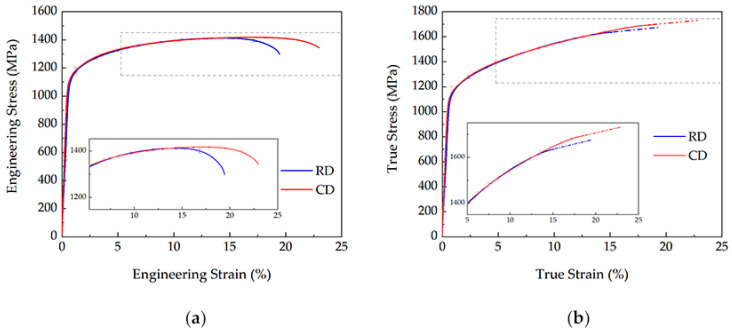
The engineering (**a**) and (**b**) true stress–strain curves of the tensile test along CD and RD.

The ex situ tensile stress–strain curves of the specimens along CD and RD are shown in Figure 3. Unlike those reported in reference [30,31], the tensile properties along the two directions approach nearly. This minor mechanical variation appears to be explained by a close microstructure in two directions, with a nonfibrous secondary phase and random grain orientation distribution. It is worth notice that the principle of volume invariance becomes less convincing when the specimen starts to neck. The corresponding sections of true stress–strain curves were interpolated as straight dashed lines.

**Figure 4:**
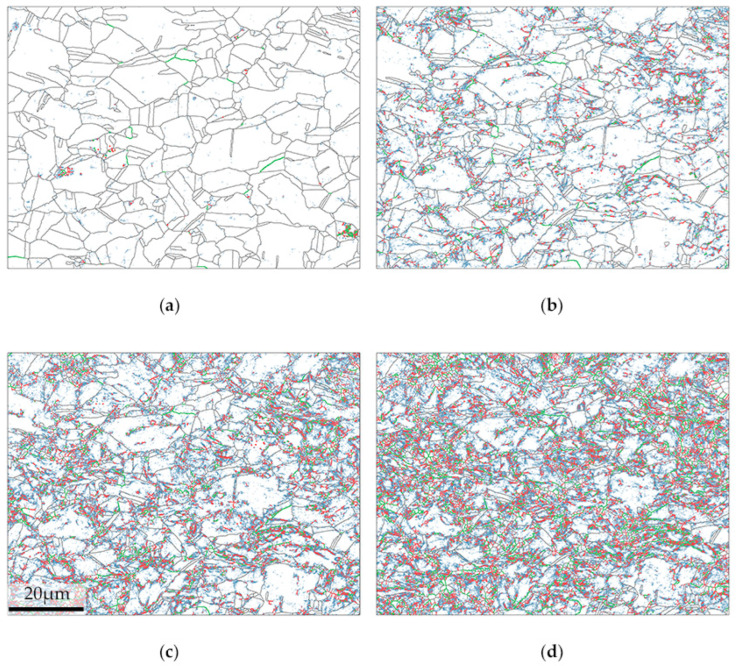
Grain boundary distribution and evolution during the in situ tensile test of CD, where blue, red and green lines represent grain boundary misorientation angles between 1–2°, 2–5° and 5–15°, respectively. Grain boundary misorientation angles larger than 15° are shown in black. EBSD was performed at strains of (**a**) 0%, (**b**) 12%, (**c**) 18% and (**d**) 24%.

In order to understand the microstructure evolution during the tensile test, an in situ tensile test was performed on the sample sliced along the CD. The evolution of grain boundary misorientation from 1–15° during the in situ tensile test on the specimen is presented in Figure 4. Before deformation, the grain boundaries mostly consisted of grain boundaries with angles larger than 15°. Low-angle grain boundaries (LAGBs) were rare at the initial state of the in situ tensile test. As the plastic deformation increased, the length of the grain boundary angle under 15° grew sharply. It can be obvious that in the early stage of deformation, regions with small grain size shows a tendency to develop LAGBs, and regions in the vicinity of these small grains have an accumulation of 2–15° grain boundaries. The LAGBs are largely consist of grain boundaries with angles between 1–2° when the mechanical strain is 12%. When the mechanical strain approached 24%, the grain boundary angle between 5–15° accumulated and got interconnected with each other. The grains whose grain size was relatively larger and equiaxed manifested themselves as a rather slow accumulation of LAGBs. Larger grains seem to be less prone to the buildup of LAGBs. Larger grains with a rather long aspect ratio appear to be more prone to LAGB accumulation than their peers except twins.

**Figure 5:**
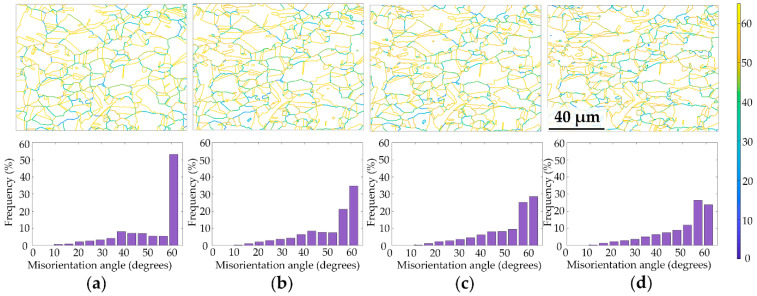
Evolution of the misorientation angle la rger than 15° at strains of (**a**) 0%, (**b**) 12%, (**c**) 18% and (**d**) 24%.

The grain boundary evolution of angles larger than 15° is presented in Figure 5. The sample mainly consists of twin boundaries with misorientation around 60°. According to the bar chart, the frequency of misorientation 60° reduced during the plastic deformation. The grain boundary frequency is relatively steady with a misorientation angle between 15–50°, while the angle between 50–60° gradually increases. Despite the fact that the field of view varied to some extent (prolonging in the horizontal direction and shortening in the vertical) during the tensile test, the frequency increase of 50–60° suggests a trend of misorientation enlargement.

**Figure 6:**
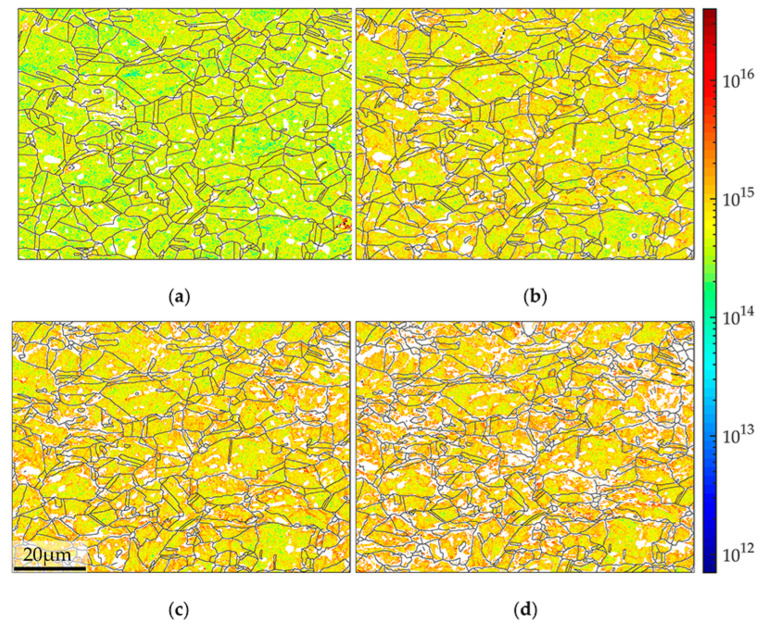
The spatial distribution and density of geometrically necessary dislocation density at strains of (**a**) 0% (**b**) 12% (**c**) 18% and (**d**) 24%. Units, m^−2^.

In an attempt to observe the interactions between dislocations and boundaries, the mapping of geometrically necessary dislocation (GND) density is evaluated. For the two-dimensional information EBSD obtained, there exists a lack of curvature tensor from z axis. Such deficiency is fitted with crystal dislocation energy. The calculation basis can be found in Wilkinson and Arsenlis’s work [32,33]. The calculation process was referred to the work of Pantleon and Yoo [34,35] in solving Nye’s [36] dislocation density tensor. The calculated spatial distribution and density of GND density is shown in Figure 6. Although the amount of collected EBSD points suffered losses from ascendant deformation, the aggregation of GND can still be recognized away from larger grains, relative to surrounding small grains. These “small” grains are geometrically segmented by random dispersed twins, which may foster the accumulation of GND through the barrier effect [37]. The boundaries between secondary phase (refer to white area in Figure 6a) and the matrix were also gathered by GND. GND gathering within grains, especially in larger grains, were caused by secondary phase blocking of dislocation movement. The concentration level of GND around secondary phase is comparatively lower than small grain region. In Figure 6c, the EBSD data loss increased due to the severity of plastic deformation, especially in grain boundary section. In Figure 6d, the EBSD data of “small” grain boundary bore great loss together with GND density information. However, the GND gathering around secondary phase within “larger” grains bore less losses, which may explain the high GND density within grains.

**Figure 7:**
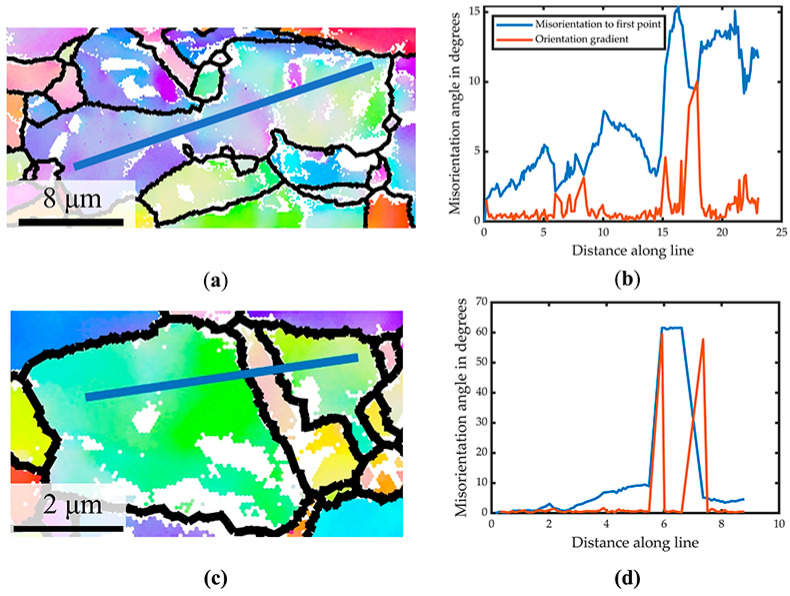
(**a**,**c**) Profile on GOI. (**b**,**d**) Misorientation to the first point (in blue) and orientation gradient (in orange) along the profile.

To further investigate the intragranular deformation of the in situ tensile process, several grains of interest (GOI) were chosen. As shown in Figure 7a, the misorientation distribution along the line across the twin showed a modest rise. This could point to the role of barrier effect twins in plastic deformation. Figure 7b illustrates a rather inhomogeneous deformation. In contrast to Figure 7a, where the misorientation peak is less than 10° near twins, the misorientation fluctuates up to 14° along the major axis. The orientation of the grains with relatively long elliptical principal axes changes dramatically over the curve seen in Figure 7b.

**Figure 8:**
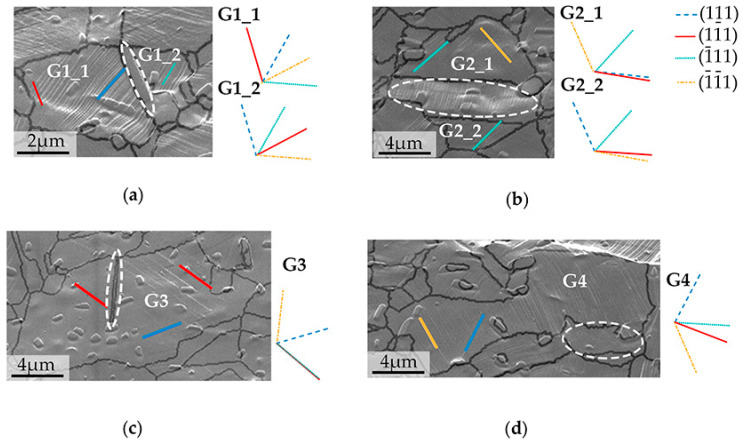
Slip systems analysis on GOI. SEM of activated slip systems and prediction traces based on EBSD data on (**a**) G1_1, G1_2, (**b**) G2_1, G2_2, (**c**) G3 and (**d**) G4. Twins in white dashed oval.

To further study the activation of slip systems, slip trace analysis was performed on several grains. The grain boundary was superimposed with the EBSD grain boundary data onto the SEM-captured surface microstructure in Figure 8. The slip traces are thought to be the intersections where activated slip systems meet the surface. If the junction line of two slip systems occurs on or near the specimen surface, the anticipated slip trace would be too close to separate, as shown by the predicted green line ($\bar{1}$11) and the red line (1$\bar{1}$1) in Figure 8c. The angle formed by these two traces is theoretically calculated to be 8.89 × 10^−5^ degrees. For a significantly smaller SF, the probability of ($\bar{1}$11) activation (green line) is ruled out (maximum 0.1588). A further identification approach for activated slip system is well established by Chen and Daly [38]. All the traces can be specifically identified by the illustrations. The intersecting lines between the four {111} slip planes and the observe surface are shown by the lines next to the SEM-captured surface. The traces obtained from SEM are very much in line with the {111} predicted traces.

The Schmid factor is a metric that describes the tendency of a grain’s slip system to activate under certain stress conditions. The Schmid factor m, can be calculated using the following Equation (1).

(1) m=cosφcosλ

where the angle between the slip direction and loading direction is φ and the angle between the slip plane normal and loading direction is λ. Schmid factor works as a geometry component of the resolved shear stress calculation as Equation (2).

(2) $$\tau_{\mathrm{rss}}{=\sigma }_{\mathrm{app}}m$$

where σ_rss_ is the applied stress. Under the same applied stress, the resolved shear stress τ_rss_ is elevated by larger Schmid factor m, which increases the likelihood of slip system activation.

For further investigation, the Schmid factor (SF) was calculated, as given in Table 1, to confirm the specific activated slip system. It is generally assumed that the slip system with the highest SF tends to be activated during mechanical deformation. The maximum and secondary Schmid factors (S_M_ and S_S_), the Schmid factor (SF) difference ratio between S_M_ and S_S_, and (S_M_ − S_S_)/S_M_ were calculated to determine the tendency of activation of the two slip systems. A comparatively small (S_M_ − S_S_)/S_M_ often implies the possibility of the activation of slip systems with maximum and secondary SF within one grain. Interestingly, in Figure 8a, G1_1 grain and G1_2 grain with close (S_M_ − S_S_)/S_M_ (10.94% and 10.82%, respectively) reveal different activation behaviors. The G1_1 grain activated the (111) slip plane (blue line), and a shallow trace (1–11) developed at the left end of the grain. With no other traces found, the G1_2 grain shows a (−111) slip plane activated (in green).

The same pattern may be seen in Figure 8b, where the twins are parallel to the tensile direction. Despite the fact that the same slip plane was determined to be ranked 1st and 2nd among the slip systems, the trace nevertheless suggests that the ($\bar{1}$$\bar{1}$1) [011] rated third slip system was activated (orange line).

This occurrence is not an outlier in the sample. The ranked 3rd (blue line) slip system was activated in Figure 8c. The SF difference ratio was calculated to be 37.58%. There is a need to mention that in Figure 8d, the twin boundary indexing is declined by the nearing large secondary phase. Grains in Figure 8c,d can somewhat be divided into two portions by twins, and the number of slip systems activated in these two parts varies. The microstructure acquired by SEM was collected at the early stage of deformation (yielding), and the ranked 3rd slip system on the right side was triggered before the left part, where the ranked 1st slip system was activated. In contrast, the grains in Figure 8d activated two slip systems, albeit having the identical feature of different numbers of slip system activations. The calculated (S_M_ − S_S_)/S_M_ for these two slip systems is 2.20%, which is a strong indication for two slip system activation. The slip system activation of these GOI shows that most grains conform to the Schmid law. The abnormal activations of slip systems were occurred in the vicinity of twins. The secondary phases were well dispersed, and there was no uncommon slip system activation neighbored them in this study. Most secondary phases broke along the slip system traces of the matrix, such as in Figure 8a, or show no impact on slip trace directions. We may conclude the effect of the secondary phase on abnormal slip system activation is much less important than the existence of twins. The twins may hinder or promote the slip system activation, thus in Figure 8b,c, ranked 3rd slip was promoted and in Figure 8d the right region with a close Schmid factor difference ratio of 2.20%, the 2nd slip system activation was hindered.

**Figure 9:**
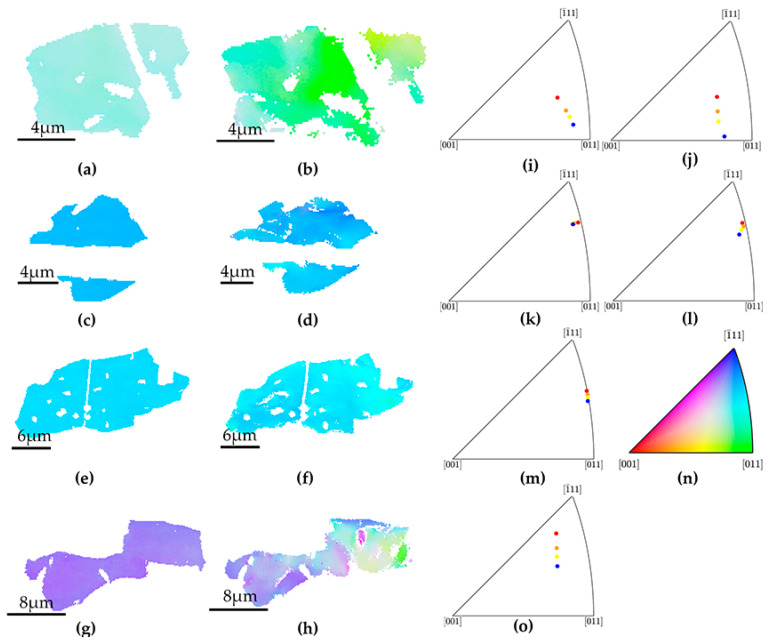
EBSD mapping of G1_1, G1_2 under strain (**a**) 0%, (**b**) 18%, G2_1, G2_2, under strain (**c**) 0%, (**d**) 18%, and G3, G4 under strain (**e**,**g**) 0%, (**f**,**h**) 18%. Mean orientation evolution in sequence of 0% (red), 12% (orange), 18% (yellow), 24% (blue), (**i**) G1_1, (**j**) G1_2 (**k**) G2_1, (**l**) G2_2, (**m**) G3 and (**o**) G4. (**n**) IPF color key.

A qualitative rotation direction of grains can be observed through the dot-to-dot direction, and the rotation angle can be directly perceived by the gap among different deformation points. Different slip system activation further leads to different grain rotation behavior within grains. In G1_1 and G2_1, two slip systems were activated, the rotation direction is rather different from G1_2 and G2_2, whose initial orientation is close. The rotation angle of G1, G2 pairs is close. In larger grains where GND accumulation is less severe, G4, the right region where single slip system activated experienced a great extent of rotation. Only one slip system activation was realized by the twin hinder effect on the ranked 2nd slip system, thus this part of the grain rotated heavily as illustrated in the EBSD map in Figure 9h. The part where two slip systems are activated rotates towards [$\bar{1}$11], while the rest rotates towards [011]. The different rotation extent between G3 and G4 may derives from higher content of secondary phase and surrounding twin configurations in G3.

## 4. Conclusions

In this study, conventional tensile tests and in situ tensile tests coupled with EBSD were applied to GH4169 rings, and the effects of ring rolling on the microstructures and mechanical properties along the circumferential direction and radial direction of the produced rings were studied. The conclusions are as follows:

1. The microstructure of the ring-rolled GH4169 alloy is evenly distributed in CD and RD directions, and the orientation distribution is random. In addition, the microstructure in both directions presents a large number of twin characteristics;
2. The tensile properties along the circumferential direction and radial direction show a close stress–strain curve in the conventional tensile tests. The ultimate tensile strength (UTS) values in RD and CD are 1422 and 1418 MPa, respectively, and the elongation (EL) values are 18.64 and 22.92%, respectively;
3. During the in situ tensile test, low-angle grain boundaries show a tendency to distribute in regions neighboring grains with relatively small sizes;
4. In the grain interior, the traces of most grains show good agreement with Schmid’s law. Several traces of grains featuring twins disobey the Schmid’s law. The interplay between secondary phase and slip system activation is less important compared to twins, which promote or hinder the slip system activation. The behavior of the slip system further influenced the rotation activity of the grains. Further study should focus mainly on the interactions between the matrix and the twins.

## Figures and Tables

**Table 1:** Schmid factor of different slip systems in GOI.

Grains of Interest	Slip System	Schmid Factor	Grains of Interest	Slip System	Schmid Factor
G1_1	(111) [ 0 $\bar{1}$ 1]	**0.4523**	G2_1	( $\bar{1}$ 11) [01 $\bar{1}$ ]	**0.4564**
	(111) [ $\bar{1}$ 10]	**0.4028**		( $\bar{1}$ 11) [ $\bar{1}$ $\bar{1}$ 0]	**0.4040**
	(1 $\bar{1}$ 1) [0 $\bar{1}$ $\bar{1}$ ]	**0.3351**		( $\bar{1}$ $\bar{1}$ 1) [011]	**0.3805**
G1_2	( $\bar{1}$ 11) [101]	**0.4517**	G2_2	( $\bar{1}$ 11) [ $\bar{1}$ $\bar{1}$ 0]	**0.4587**
	( $\bar{1}$ 11) [ $\bar{1}$ $\bar{1}$ 0]	**0.4028**		( $\bar{1}$ 11) [101]	**0.4002**
	(111) [10 $\bar{1}$ ]	**0.3328**		(111) [ $\bar{1}$ 10]	**0.3857**
G3	(1 $\bar{1}$ 1) [110]	**0.4037**	G4	( $\bar{1}$ $\bar{1}$ 1) [ $\bar{1}$ 0 $\bar{1}$ ]	**0.4448**
	(1 $\bar{1}$ 1) [ $\bar{1}$ 01]	**0.3710**		(111) [10 $\bar{1}$ ]	**0.4350**
	(111) [0 $\bar{1}$ 1]	**0.2520**		( $\bar{1}$ $\bar{1}$ 1) [011]	**0.3759**

## Data Availability

The data are available upon request.
